# Sleep Diagnostics for Home Monitoring of Sleep Apnea Patients

**DOI:** 10.3389/fdgth.2021.685766

**Published:** 2021-06-15

**Authors:** Dorien Huysmans, Pascal Borzée, Bertien Buyse, Dries Testelmans, Sabine Van Huffel, Carolina Varon

**Affiliations:** ^1^STADIUS Center for Dynamical Systems, Signal Processing and Data Analytics, Department of Electrical Engineering (ESAT), KU Leuven, Leuven, Belgium; ^2^Department of Pneumology, UZ Leuven, Leuven, Belgium; ^3^e-Media Research Lab, Department of Electrical Engineering (ESAT), KU Leuven, Leuven, Belgium

**Keywords:** sleep, sleep apnea, unobtrusive sensor, ECG, respiration, convolutional neural network, home monitoring

## Abstract

**Objectives:** Sleep time information is essential for monitoring of obstructive sleep apnea (OSA), as the severity assessment depends on the number of breathing disturbances per hour of sleep. However, clinical procedures for sleep monitoring rely on numerous uncomfortable sensors, which could affect sleeping patterns. Therefore, an automated method to identify sleep intervals from unobtrusive data is required. However, most unobtrusive sensors suffer from data loss and sensitivity to movement artifacts. Thus, current sleep detection methods are inadequate, as these require long intervals of good quality. Moreover, sleep monitoring of OSA patients is often less reliable due to heart rate disturbances, movement and sleep fragmentation. The primary aim was to develop a sleep-wake classifier for sleep time estimation of suspected OSA patients, based on single short-term segments of their cardiac and respiratory signals. The secondary aim was to define metrics to detect OSA patients directly from their predicted sleep-wake pattern and prioritize them for clinical diagnosis.

**Methods:** This study used a dataset of 183 suspected OSA patients, of which 36 test subjects. First, a convolutional neural network was designed for sleep-wake classification based on healthier patients (AHI < 10). It employed single 30 s epochs of electrocardiograms and respiratory inductance plethysmograms. Sleep information and Total Sleep Time (TST) was derived for all patients using the short-term segments. Next, OSA patients were detected based on the average confidence of sleep predictions and the percentage of sleep-wake transitions in the predicted sleep architecture.

**Results:** Sleep-wake classification on healthy, mild and moderate patients resulted in moderate κ scores of 0.51, 0.49, and 0.48, respectively. However, TST estimates decreased in accuracy with increasing AHI. Nevertheless, severe patients were detected with a sensitivity of 78% and specificity of 89%, and prioritized for clinical diagnosis. As such, their inaccurate TST estimate becomes irrelevant. Excluding detected OSA patients resulted in an overall estimated TST with a mean bias error of 21.9 (± 55.7) min and Pearson correlation of 0.74 to the reference.

**Conclusion:** The presented framework offered a realistic tool for unobtrusive sleep monitoring of suspected OSA patients. Moreover, it enabled fast prioritization of severe patients for clinical diagnosis.

## 1. Introduction

Obstructive Sleep Apnea (OSA) is the most common sleep related breathing disorder. It is characterized by events of breathing disturbances causing hypoxemia, intrathoracic pressure changes and arousals from sleep. Consequently, OSA is an acknowledged risk factor for excessive daytime sleepiness, hypertension and cardiovascular diseases (1). As OSA is closely associated with obesity and advancing age, the prevalence is expected to further increase (2). Nevertheless, many patients remain undiagnosed. One of the reasons is the limited hospital capacity for performing polysomnography (PSG) (3). Furthermore, the clinical diagnostic procedure poses a high level of discomfort for the patient. Therefore, it is desired to identify OSA patients at risk with unobtrusive sensors at home, allowing a comfortable sleeping environment and follow up over multiple nights. Clinically, the severity of sleep apnea is assessed by the Apnea-Hypopnea Index (AHI), which is the number of respiratory events (apneas, hypopneas and respiratory effort-related arousals) per hour of sleep. The events are annotated based on the patient's airflow and oxygen saturation (4). A patient is then categorized as not suffering from OSA if 0 ⩽ AHI < 5, mild OSA if 5 ⩽ AHI < 15 with presence of symptoms, moderate OSA if 15 ⩽ AHI < 30 or severe OSA if AHI ⩾ 30 (5). The calculation of this AHI requires the quantification of the hours of sleep, i.e., Total Sleep Time (TST). In fact, there are five sleep stages defined by the American Academy of Sleep Medicine, which are Wakefulness, Rapid Eye Movement sleep (REM sleep) and non-REM (NREM) sleep 1, 2, and 3 (respectively N1, N2, and N3) (4). Usually, stages N1 and N2 are referred to as *light* sleep and N3 as *deep* sleep. The rules for annotating sleep stages (i.e., performing sleep staging) are based on patterns and wave characteristics found in the electroencephalogram (EEG), the electrooculogram, and the submental electromyogram. The PSG records these signals, among others such as the respiratory airflow, oxygen saturation and electrocardiogram (ECG). To facilitate the sleep staging, these signals are scored in consecutive windows of 30 s, which are referred to as epochs (6). Hence, in this paper, monitoring of sleep apnea patients refers to the whole process of sleep staging, sleep time estimation and severity assessment.

Although clinical sleep staging mainly relies on EEG analysis, many emerging unobtrusive sensor technologies for sleep monitoring are based on cardiac and respiratory signals. Consequently, the development of novel algorithms for automated sleep staging based on these unobtrusive signals is an active topic of research. The following studies developed specific sleep staging algorithms for OSA patients based on cardiac and respiratory information. Often, feature-based approaches were implemented to differentiate between sleep stages when expert knowledge was available (7–10). This implied a disadvantage of the method as prior knowledge was required to find appropriate features. Another disadvantage was the extensive data processing needed to perform accurate feature extraction. To alleviate the manual feature extraction, a deep learning network can be developed, as done by (11). The network required an input sequence of 100 × 30 s epochs and obtained good performance results for classifying the five sleep stages. These algorithms by previously mentioned authors required long signal segments surrounding a 30 s (or 60 s) epoch as an input for the epoch's sleep stage classification. As such, these longer segments provided contextual information to improve classification performance. However, long intervals of good quality are in reality not available as unobtrusive sensors are very sensitive to movement artifacts. In addition, OSA patients often show more movements during their sleep compared to healthy subjects. Therefore, the required algorithm input should consist of single and independent signal epochs, to alleviate the requirement of successive good quality segments. However, state-of-the-art sleep staging algorithms rarely take into account the potential data loss and distortion of unobtrusive sensors. Malik et al. (12) did perform a two-class sleep-wake classification with an input consisting of single 30 s epochs, or longer sequences. They solely used the instantaneous heart rate (IHR) (i.e., tachograms) and a one-dimensional convolutional neural network (1D CNN). However, the method was only applied on healthy subjects and the performance on 30 s epochs was insufficient. Also in the study of (13), a sleep-wake classifier was developed with 30 s epochs, for healthy to mild OSA patients and based on the 1D CNN of (12). A difference with the classifier of (12) was that respiratory inductance plethysmography (RIP) signals were added to improve performance. Moreover, the use of tachograms allowed a straight-forward application of other sensors capturing the beat-to-beat variability. As such, the CNN was preliminarily tested with recordings from unobtrusive capacitively coupled ECG. However, the study was based on a limited dataset.

Additionally, in OSA patients, heart rate disturbances and sleep fragmentation complicates algorithm design and validation (14, 15). The complexity and validation issue are related to the increase of the uncertainty in clinical sleep staging with the AHI of a patient. It is partially a consequence of the restrictions posed by the scoring rules, as defined in (4). For example, patients can pass through two or even three different sleep stages during a 30 s interval, although sleep stages are annotated per epoch of 30 s. Also micro-sleeps or micro-awakenings of a few seconds will not be annotated. Additionally, apneic events can only be scored if they last at least 10 s. State-of-the-art non-EEG sleep staging algorithms are aware of the decrease in prediction performance for a patient population, however the problem is not mitigated (8, 16). Therefore, it is desired to detect OSA patients with complex sleep architectures, as they would receive less reliable sleep-wake predictions and can be prioritized for a clinical PSG.

The primary aim of this actual work is to reliably estimate TST for healthy subjects as well as the whole range of OSA patients, based on PSG signals which could be acquired unobtrusively. This means the TST is estimated based on single short-term segments, as unobtrusive data likely includes artifacts and data loss. Therefore, this study proposes a sleep-wake classifier based on (13), which can handle data acquired by unobtrusive sensors. For this, the approach proposed here has a preprocessing phase based on single 30 s segments, as opposed to the previous algorithm, which makes it more usable for future application on unobtrusive data. Furthermore, the robustness of the network is verified by training the CNN model multiple times using a variation in training and validation set and by comparing the performance of each model on a test set. This is in contrast with the application of a fixed training and validation set. In addition, the network is tested on the whole range of OSA patients, instead of only healthy and mild OSA patients. The secondary aim is to assess the applicability of the classifier's outcome for detection of OSA patients, who would receive less reliable sleep-wake predictions. The TST estimates of these patients would be less accurate, but they can be directly prioritized for a clinical diagnostic test. Thus, the relationships between a patient's classification outcome and its OSA severity is analyzed. As the sleep-wake network is trained on healthy subjects and mild OSA patients, a relatively small amount of apneic events is included in the training set. Thus, a first hypothesis is that the CNN classifier will exhibit uncertain sleep-wake predictions in the presence of apneic events. The second hypothesis is that more transitions from sleep to wake and vice versa occur in the predicted sleep pattern of OSA patients, also caused by apneic events. Hence, this study addresses the need for a sleep monitoring framework that accommodates signals acquired by unobtrusive sensors, as it takes into account data losses through the analysis of single short-term segments. Furthermore, the framework investigates how the predicted sleep architecture of OSA patients and the decrease in reliability can be applied to detect these patients, and increase overall sleep monitoring performance.

## 2. Materials and Methods

**Figure 1:**
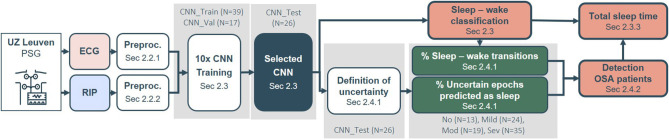
Framework pipeline from polysomnography (PSG) data to Total Sleep Time (TST) estimation and detection of OSA patients. First, the electrocardiogram (ECG) and respiratory inductance plethysmogram (RIP) data were preprocessed (section 2.2). The classifier's architecture was based on a convolutional neural network (CNN). Its training procedure and derived TST outcome is reported in section 2.3. The relation between uncertainties and sleep-wake transitions in a patient's prediction and the obstructive sleep apnea (OSA) severity were studied in section 2.4.1. These relations were subsequently applied for detection of OSA patients (section 2.4.2). The gray boxes indicate the datasets used for parameter optimization or selection.

This study is organized as illustrated in Figure 1. First, the different datasets and their demographics and sleep information are described in section 2.1. Section 2.2 presents the preprocessing methodology of ECG and RIP data. The classifier's architecture, its training procedure and the derivation of the TST are described in section 2.3. Furthermore, section 2.4 studies the link between a patient's sleep-wake prediction and its OSA severity in order to detect OSA patients.

### 2.1. Datasets

The dataset comprised 183 patients who were referred to the sleep laboratory of the University Hospitals Leuven (UZ Leuven, Belgium) for a diagnostic PSG. The B3IP device from Medatec (Haillot, Belgium) served as polysomnograph and provided data from the built-in ECG (SPES electrodes) and built-in thoracic RIP (SleepSense belts) (17). Medatec Brainnet Winacq 5.0 was the acquisition software and Medatec Brainnet Winrel 5.0 the analyzing software. A clinical sleep expert annotated the sleep stages and apneic events according to the AASM 2012 scoring rules (4). The collection of data was approved by the ethical committee of UZ Leuven (S60319) and all patients signed an informed consent. From the full dataset, 36 patients were left out as an independent, unseen dataset for validation of sleep-wake classification, TST estimation and detection of OSA patients. These patients were part of an additional data collection later in time, complying with the same ethical standards. The remaining patients were split into subsets for different purposes, as described in section 2.3.2. The overview of the different subdatasets can be found in Table 1. Figure 1 indicates which datasets were applied for parameter optimization or model selection.

**Table 1:** Demographic and clinical information of the study datasets.

**Dataset**	***N***	**AHI (1/h)**	**Average Nr. of Epochs (% of Total Nr. Epochs)**				
	**(AHI <5)**	**Mean (SD)**	**N3**	**N2**	**N1**	**REM**	**Wake**
*CNN_Train*	39 (13)	5.9 (2.2)	230 (22)	412 (40)	38 (4)	172 (17)	175 (17)
*CNN_Val*	17 (9)	5.1 (2.6)	253 (24)	393 (36)	39 (4)	159 (15)	236 (22)
*CNN_Test*	26 (13)	4.8 (2.6)	221 (22)	416 (40)	44 (4)	182 (17)	168 (17)
No	13	2.2 (1.3)	233 (22)	444 (42)	46 (4)	196 (18)	147 (14)
Mild	24	8.7 (2.9)	186 (19)	388 (39)	50 (5)	148 (15)	235 (23)
Mod	19	20.4 (4.3)	189 (19)	391 (38)	55 (5)	139 (14)	239 (24)
Sev	35	61.9 (20.1)	120 (12)	383 (39)	139 (14)	111 (12)	236 (24)
Test	36	38.6 (29.6)	112 (11)	406 (39)	104 (11)	128 (12)	281 (27)

*The whole dataset comprised 183 patients. Training of the sleep-wake classifier was performed on 39 patients (CNN_Train) and validated on 17 patients (CNN_Val), all with AHI <10. The trained classifier was tested with CNN_Test. In addition, dataset CNN_Test was merged with patients with higher AHI and split again according to OSA severity in the subsets No, Mild, Mod, and Sev. Out of 183 patients, 36 patients were left out as an unseen dataset Test for independent validation of sleep-wake classification, total sleep time estimation and detection of OSA patients*.

*N, Number of subjects in dataset, with number of No OSA indicated in brackets. AHI, Apnea-Hypopnea Index*.

*No: AHI < 5, Mild: 5 ⩽ AHI < 15, Mod: 15 ⩽ AHI < 30, Sev: AHI ⩾ 30*.

### 2.2. Data Preprocessing

The sleep-wake classification network was developed based on full-night recordings of ECG and RIP, extracted from the PSG. The preprocessing steps took into account the application on unobtrusive, movement-sensitive sensor recordings, with frequent episodes of insufficient quality. As such, the full signal was first segmented into non-overlapping windows of 30 s and preprocessing was performed on these individual segments.

#### 2.2.1. ECG

First, R-peak detection was performed on 30 s segments, with the method proposed by (18). Segments with less than 15 detected R-peaks were discarded. From the remaining segments, the IHR was derived and expressed in beats per minute. The unevenly sampled IHR data points were interpolated at 4 Hz by a piecewise cubic hermite interpolating polynomial, resulting in segments of 120 samples. To avoid border problems during interpolation, the first and last beat of the segment were shifted in time. The first beat time was calculated by subtracting the mean value of the second and third interbeat interval from the second beat time. Similarly, the last beat time was calculated by adding the mean value of the second and third last interbeat interval to the second last beat time. Next, outliers were identified whenever the IHR value was outside the range of 40–180 beats per minute, or outside the segment's median value ± 20 beats per minute, or outside the segment's median value ± [3 × the segment's standard deviation (SD)]. The first condition were physiological boundaries. The second and third were defined empirically using visual inspection and logical values. Next, the outliers were indicated with NaN. The NaN interval was corrected as long as the duration of subsequent NaNs was smaller or equal to 10 samples (i.e., 2.5 s). This NaN gap was filled by mirroring the values preceding the gap (19). Outlier correction was important to not discard epochs with minor artifacts and preserve a maximal number of epochs. Finally, the interpolated values of remaining segments were concatenated and the overall median for each subject was subtracted from every segment. In this way, inter-subject variability was removed but the inter-sleep stage variability retained. As a neural network cannot process NaN values, every segment with remaining NaN values was discarded.

#### 2.2.2. RIP

The segments of the RIP signal were bandpass filtered at [0.04, 2] Hz and downsampled to 4 Hz by spline interpolation, resulting in segments of 120 samples. Then, the median and SD value of every segment was considered. As such, every patient recording had a distribution of median values and one of SD values. Next, every segment was normalized by subtraction with the 50th percentile of the median values and dividing by the 50th percentile of the SD values, to reduce the influence of respiratory artifacts. This was followed by the subtraction of the individual median per segment. Segments discarded after ECG preprocessing as they contained remaining NaN values, were also discarded from the RIP data. Remaining epochs, i.e., without NaNs, were fed to the neural network.

### 2.3. Sleep-Wake Classification

#### 2.3.1. Neural Network Architecture

**Figure 2:**
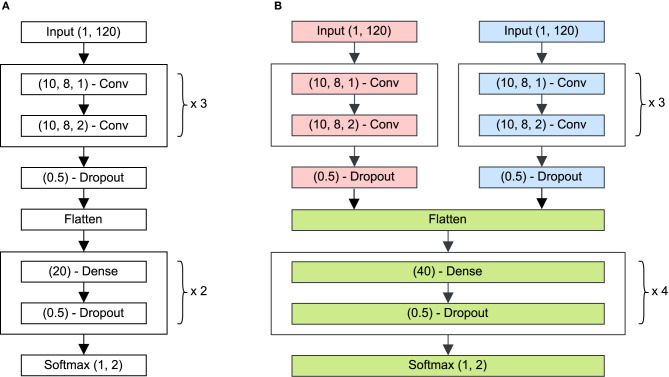
**(A)** Illustration of the network architecture used for the cardiac and the respiratory signals separately. This CNN extracted characteristics from the input epoch with length 120 samples. The layer (*f, k, s*) − *Conv* indicates a layer with *f* filters, kernel size *l* and stride *s*, and an activation of type *ReLu*. After the convolutional block, dense layers, (*n*) − *Dense*, with *n* neurons were included. A third type were dropout layers, (*d%*) − *Dropout*, where *d%* = 50% of the weights were equal to zero in every training step to avoid overfitting. The output layer is a softmax layer, *Softmax(1,c)*, delivering posterior class probabilities for every one of the *c* = 2 classes, where class 0 represented Sleep and class 1 Wake. **(B)** Architecture of the combined deep learning network, consisting of a cardiac CNN (red), a respiratory CNN (blue) and a combined dense network (green). During training, the weights of convolutional branches were frozen, while the dense layers were optimized.

The neural network consisted of a convolutional part for feature representation and a dense part for classification (see Figure 2). Two separate unimodal networks were first optimized using the cardiac or respiratory signal, based on (12). After training, the convolutional layers of these networks were combined into a multimodal network, retaining the weights of these layers. Only the dense layers of the multimodal network were optimized using training. All networks consisted of four types of layers. The convolutional layers were defined as (*f, k, s*) − *Conv*, with a depth *f*, a kernel size *k*, a stride *s* and an activation of type *ReLu*. After the convolutional block, dense layers, (*n*) − *Dense*, with *n* neurons were included. A third type were dropout layers, (*d%*) − *Dropout*, where *d%* = 50% of the nodes were set to zero in every training step to avoid overfitting (20). The output layer is a softmax layer, *Softmax*(1, *c*), delivering posterior class probabilities for every one of the *c* = 2 classes, where class 0 represented *Sleep* and class 1 *Wake*. As an optimization scheme, *Adam* was chosen, which uses an adaptive learning rate for weight updates instead of a fixed rate (21). The network trained with balanced and shuffled batches of sixteen non-sequential epochs. Balancing was achieved by over-sampling classes, such that every batch contained on average an equal number of samples of every class. The threshold of posterior class probability for classification was set at 0.5, thus assigning a segment to class *Wake* if *p*_*class*_⩾0.5.

#### 2.3.2. Neural Network Training and Selection

Training of the network was performed on 56 patients from UZ Leuven with a low AHI (i.e., AHI < 10), so that the network purely learned patterns of sleep or wake and not to recognize apneic events for classification. Moreover, patients with higher OSA severity have stronger physiological dynamics, which may block the learning process of typical sleep patterns. The training dataset was randomly split into a subset using 70% (*N* = 39) of the patients for weight training of the neural network (*CNN_Train*) and 30% (*N* = 17) for validation during training (*CNN_Val*), with N the number of subjects. The subdivision changed ten times, using a different seed for randomization, to train and validate ten models. The same ten seeds were used for both the unimodal ECG and RIP networks as well as for the multimodal network. The final multimodal model was selected based on the highest Cohen's Kappa score (κ) obtained using the fixed (i.e., non-randomized) set *CNN_Test*. The κ score is a measure of inter-rater agreement, while compensating for the degree of agreement expected by chance. It ranges from –1 (total disagreement) through 0 (random classification) to 1 (total agreement). The interpretation of κ, however, varies among different studies (22).

In addition, the patients of dataset *CNN_Test* were merged with patients with higher AHI and split again according to clinical OSA categories in the subsets *No, Mild, Mod*, and *Sev*. As such, the selected sleep-wake classifier tested these populations with varying AHI. Finally, a Wilcoxon signed rank test verified the performance differences between the unimodal networks, and between the unimodal vs. multimodal network on the patients in *No, Mild, Mod*, and *Sev*.

#### 2.3.3. Assessment of Total Sleep Time

The TST was estimated as the total time spent asleep in minutes, for datasets *No, Mild, Mod, Sev*, and *Test*. The comparison was performed by subtracting the reference TST from the estimated TST and calculating the mean and SD of this difference. In addition, the Pearson's correlation coefficient ρ between the reference TST and estimated TST was calculated.

### 2.4. Detection of OSA Patients Based on Sleep-Wake Classifier Outcome

The secondary aim of this study was to assess the applicability of the classifier's outcome for detection of OSA patients. Therefore, the relationships between a patient's outcome of the sleep-wake classifier and its OSA severity was analyzed in section 2.4.1. These relations were used as metrics for which appropriate thresholds were required to detect OSA patients. Threshold selection was performed in section 2.4.2.

#### 2.4.1. Relations Between Sleep-Wake Classifier Outcome and OSA Severity

**Figure 3:**
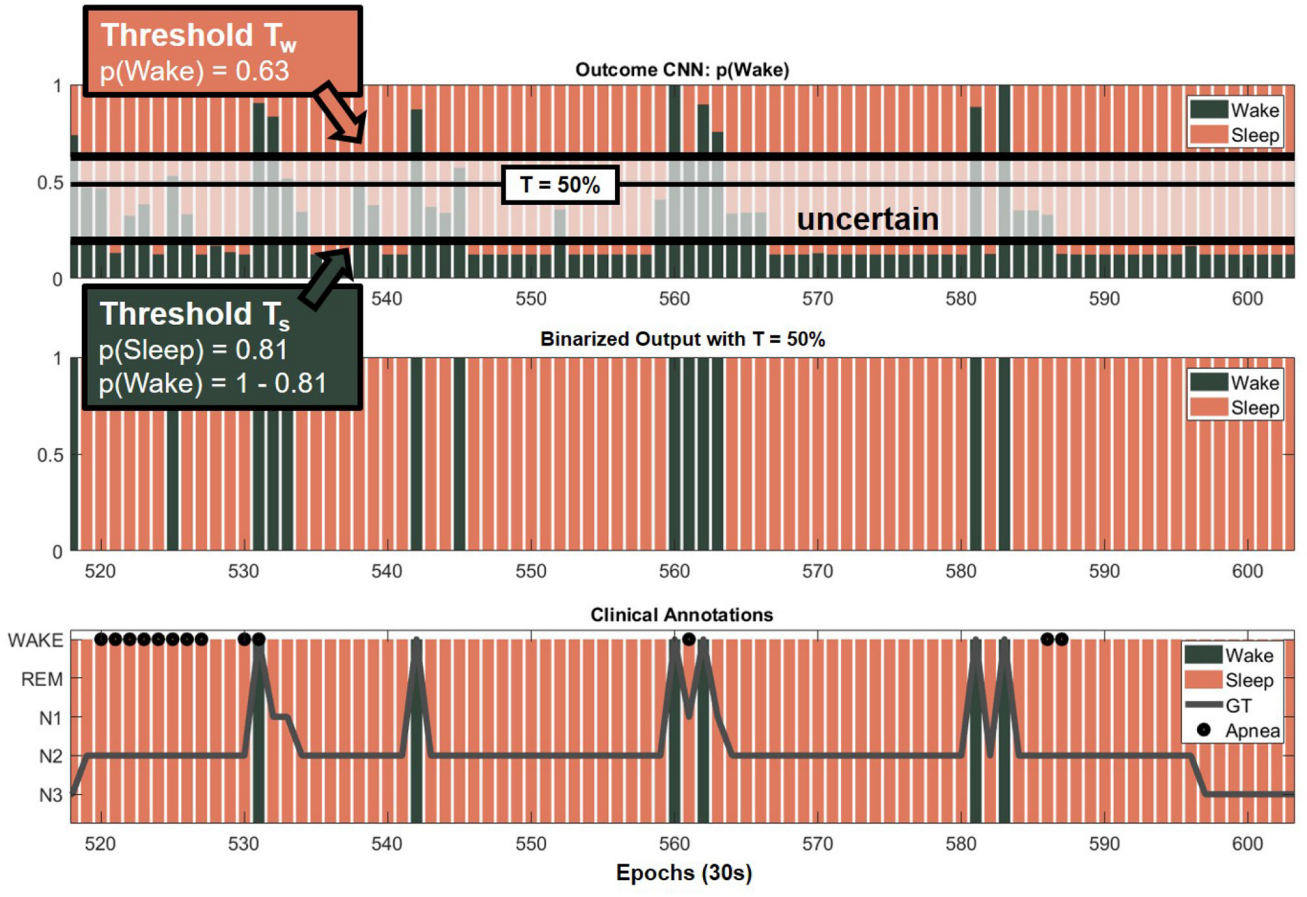
Definition of uncertain epochs. The top row represents the outcome of the CNN of each epoch as *p*(*Wake*) (green) and *p*(*Sleep*) = 1 − *p*(*Wake*) (red) (see section 2.3.1). A distinction was made between *confident* and *uncertain* predicted epochs by defining confidence thresholds. The second row shows the predicted sleep-wake classification with the default threshold at 50%. The last row shows the ground truth (GT) sleep stages which were clinically annotated.

The sleep-wake classifier network was trained on a rather healthy population (*CNN_Train* with AHI < 10), in which a relatively small amount of apneic events was present. It was hypothesized that the network output would exhibit uncertain sleep-wake predictions in the presence of apneic events, as mentioned in the introduction. Therefore, the probabilistic outcome of *CNN_Test* was further inspected to increase insight into the predictions, as explained further on and illustrated in Figure 3. The top row represented the outcome of the CNN, which was the wake probability of each epoch, i.e., p(Wake). The second row shows the predicted sleep-wake classification with the threshold for posterior class probability at 50% (see section 2.3.1). The last row showed the ground truth sleep stages, which clinicians annotated. However, as can be seen from the top row, some epochs had a p(Wake) just above 50%. Thus, the prediction of these epochs was rather uncertain. On the other hand, an epoch with a very low p(Wake), e.g., 10%, indicated an epoch which was predicted as *Sleep* with a high confidence. Based on these observations, a distinction was made between *confident* and *uncertain* predicted epochs by defining confidence thresholds (**Table 3**). The wake confidence threshold T_w_ served as the threshold for epochs predicted as *Wake*. It was the median p(Wake) of epochs predicted as *Wake* minus its SD, calculated over all subjects of *CNN_Test*. For epochs predicted as *Sleep*, the p(Sleep) = 1 − p(Wake) was considered. Thus, the sleep confidence threshold T_s_ was the median p(Sleep) of epochs predicted as *Sleep* minus its SD, calculated over all subjects of *CNN_Test*. Epochs with a p(Wake) between these margins had an uncertain prediction. These margins were applied on sets *No, Mild, Mod, Sev* and *Test*. Thus, the amount of uncertain sleep or wake predictions over the total number of predicted epochs was investigated as an indicator of apneic severity, referred to as % Uncertain Sleep Epochs and % Uncertain Wake Epochs.

In addition, the predicted sleep architecture was expected to exhibit more frequent sleep-wake transitions with increasing AHI. Reasons for this included the expected increase of sleep fragmentation with the amount of apneic events (23), the presence of micro-awakenings due to apneas and the sympathetic activation related to apneas that resemble cardiorespiratory behavior during wakefulness (15, 24). Due to the latter, the network might predict a wake epoch shortly after the occurrence of an apneic event although the patient continued sleeping. Therefore, the percentage of wake-sleep *plus* sleep-wake transitions in the prediction was examined as a second identification metric for high risk OSA patients, referred to as % Sleep Wake Transitions. More precisely, every change in the prediction from wake to sleep or vice versa was counted and divided over the total number of predicted epochs. Only remaining (i.e., without NaNs) epochs were counted.

#### 2.4.2. Detection of OSA Patients

The goal was to apply the sleep-wake classifier outcome, namely the metrics % Uncertain Sleep Epochs and % Sleep Wake Transitions, for detection of OSA patients. Firstly, to gain insight into the suitability of these metrics for patient detection, the distributions of both metrics were visualized with boxplots per OSA severity class. This was performed using the four datasets *No, Mild, Mod* and *Sev*. An upward trend of each metric with OSA severity was expected. Thus, a Kruskal–Wallis test with Bonferroni correction tested significant differences (*p* < 0.05) between OSA classes. As a patient is regarded as suffering from OSA if the AHI ⩾ 15, regardless of having symptoms, the presented method should be able to select moderate (15 ⩽ AHI < 30) and severe patients (AHI ⩾ 30). For simplicity, it was chosen that if at least one of both metrics exceeded a selected threshold, the patient was identified as being at high risk of OSA, i.e., detected positive. Therefore, ROC analysis was carried out to select a suitable OSA detection threshold for each metric. A large specificity was preferred when setting the thresholds, as this meant the identified OSA group would contain few false positives, i.e., few non-OSA patients falsely detected to have OSA. Hence, this implied the detection of patients with rather high AHI values, as opposed to AHI values close to 15 events/h. Hence, when detecting OSA patients at home using only unobtrusive cardiac and respiratory sensors, moderate and severe OSA patients could be detected with a high confidence and given prioritization for a diagnostic PSG. This procedure for detecting OSA patients was assessed on the Test data set.

## 3. Results

### 3.1. Sleep-Wake Classifier Selection and Performance

The multimodal network was trained ten times on different distributions of *CNN_Train* and *CNN_Val*. Application of these 10 networks onto *CNN_Test* resulted in moderate κ scores ranging between 0.46 and 0.51. The multimodal model with the highest κ was chosen.^1^ The weights of the convolutional layers of this chosen multimodal network were the same as the final ECG and RIP unimodal networks. Application of *CNN_Test* on the selected ECG, RIP and multimodal networks resulted in κ = 0.31, 0.46, and 0.51, respectively. In addition, the multimodal CNN tested all other datasets. Table 2 shows the resulting κ scores. Using all patients with varying AHI, the Wilcoxon signed rank test indicated significant different κ scores (*p* < 0.05) for the RIP and ECG+RIP networks compared to the ECG network, and the RIP compared to the ECG+RIP network. Next, the TST estimates were compared with the reference value for all datasets (Table 2).

**Table 2:** Sleep-wake classification of the unimodal and multimodal networks and TST estimates.

	**Data**	**κ**		**TST**		**ρ**
		**Median**	**SD**	**Mean(Δ) (min)**	**SD(Δ) (min)**	
ECG	*CNN_Test*	0.31	0.15	47	88	0.21
RIP	*CNN_Test*	0.46	0.17	52	60	0.72
	*CNN_Train*	0.53	0.16	2	48	0.83
	*CNN_Val*	0.48	0.21	10	71	0.44
	*CNN_Test*	0.51	0.13	0	50	0.78
ECG + RIP	No	**0.51**	0.14	−9	**34**	0.90
	Mild	**0.49**	0.16	−9	**65**	0.60
	Mod	**0.48**	0.17	−10	**75**	0.42
	Sev	**0.36**	0.20	26	**86**	0.56
	Test	0.32	0.18	−9.7	101	0.46

***Left**: Performance score κ is calculated with decision threshold 50%. The indicated outcomes show a similar performance for No, Mild, and Mod. **Right**: The estimated TST was compared to the TST derived from the expert annotations of the tested epochs by the mean and SD of the difference, and ρ. The outcomes indicated in bold and gray show an increase in SD(Δ) for increasing AHI. It demonstrates a decrease in reliability of the outcome*.

*TST, Total Sleep Time. Δ, (Estimated TST - Reference TST)*.

*ρ, Pearson's correlation coefficient*.

### 3.2. Uncertainty in Sleep-Wake Classifier Outcome

The probability of an epoch predicted as sleep, p(Sleep), or wake, p(Wake), are shown in Table 3. The median and SD values of p(Sleep) and p(Wake) of dataset *CNN_Test* defined the confidence thresholds for the multimodal network (see section 2.4.1). This resulted in T_s_ = 0.87 − 0.06 = 0.81 and T_w_ = 0.69 − 0.06 = 0.63. Taking these thresholds into account, the percentages of uncertain predicted sleep and wake epochs were derived and displayed in Table 3. ECG based predictions appear more difficult as the % Uncertain Sleep Epochs was highest compared to RIP and ECG+RIP. Instead, for RIP and ECG+RIP outcomes, this number increased with AHI.

**Table 3:** Uncertainty in the sleep-wake classifier outcome, averaged over individual patient outcomes.

	**Data**	**p(Sleep)**		**p(Wake)**		**% USE**			**% UWE**		
		**Med**	**SD**	**Med**	**SD**	**Med (SD)**	**NA**	**A**	**Med (SD)**	**NA**	**A**
ECG	*CNN_Test*	**0.65**	**0.01**	**0.59**	**0.03**	42 (10)	40	2	41 (8)	39	2
RIP	*CNN_Test*	**0.89**	**0.02**	**0.67**	**0.05**	37 (13)	34	2	34 (12)	32	2
	*CNN_Train*	0.87	0.09	0.69	0.06	37 (19)	33	3	40 (11)	35	3
	*CNN_Val*	0.80	0.10	0.67	0.05	51 (21)	47	3	40 (11)	37	1
	*CNN_Test*	**0.87**	**0.06**	**0.69**	**0.06**	33 (12)	31	3	40 (9)	37	2
ECG + RIP	*No*	0.87	0.02	0.69	0.07	**33 (11)**	32	1	40 (9)	38	2
	*Mild*	0.87	0.08	0.69	0.04	**39 (13)**	34	4	40 (9)	35	5
	*Mod*	0.86	0.09	0.68	0.07	**45 (17)**	31	13	41 (8)	31	7
	*Sev*	0.66	0.11	0.65	0.04	**62 (21)**	21	39	47 (9)	20	27

***Left**: Prediction probability of sleep predicted epochs [p(Sleep) = 1 − p(Wake) with p(Wake) <50%], and wake predicted epochs [p(Wake)⩾50%]. The values indicated in green were used to calculate T_s_ and red for T_w_. **Right**: Percentage of uncertain epochs predicted as sleep or wake, derived using T_s_ and T_w_. From the indicated values it can be seen that % Uncertain Sleep Epochs increased with OSA severity, supporting the usage of this metric as an indicator of apneic severity. Furthermore, a distinction was made between uncertain epochs during which an apneic event occurred (A) and those without (NA), indicated with the mean value over all subjects*.

*M, Median value; USE, Uncertain Sleep Epochs; UWE, Uncertain Wake Epochs; NA, No apneic events; A, With apneic events*.

To further investigate the origin of uncertain epochs, a distinction was made between uncertain epochs with and without apneas. For *No, Mild, Mod*, and *Sev*, the % Uncertain Sleep Epochs *with* the presence of an apneic event were, respectively, 1, 4, 13, and 39%. Thus, it was found that apneic events caused the increase in % Uncertain Sleep Epochs with AHI. The % Uncertain Sleep Epochs *without* the presence of an apneic event were, respectively, 32, 34, 31, and 21%. These values stayed rather stable over the datasets with increasing AHI, however, a clear decrease was seen for Sev. To investigate the cause of uncertainty for non-apneic epochs, the ground truth sleep stages of these uncertain epochs were extracted for *CNN_Test*. The largest portion of uncertain sleep predicted, non-apneic epochs were present during N2 and REM sleep as seen in Supplementary Table 1. On the other hand, N2 was also the most frequent sleep stage, as seen in Table 1. Therefore, the portion of uncertain non-apneic epochs per sleep stage was investigated. For this, the classes N1 and REM had the largest ratio, being 55.1 and 53.2%, respectively. However, uncertain predictions did not necessarily imply incorrect predictions. Nevertheless, classes N1 and REM also had the largest ratio of uncertain non-apneic epochs which were wrongly predicted, respectively 9.1 and 4.3%. These results can be found in more detail in Supplementary Table 2.

### 3.3. Detection of OSA Patients

**Figure 4:**
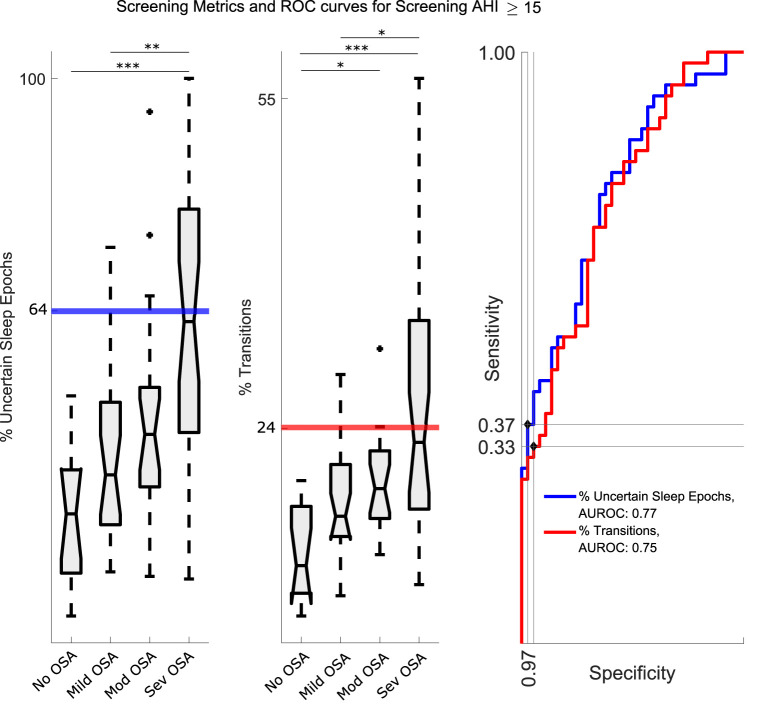
**Left and Middle:** The distribution of % Uncertain Sleep Epochs and % Sleep Wake Transitions for *No, Mild, Mod* and *Sev*. The increasing trends supported the application of the metrics for detection of OSA patient (i.e., with an AHI⩾15). The thick lines represent the chosen thresholds. The level of statistical significance is marked with one asterisk (*) if *p* < 0.05, two (**) if *p* < 0.01, and three (***) if *p* < 0.001. **Right:** Separate ROC curves for both metrics. Indicated dots were the chosen operating points and the corresponding thresholds are indicated on the left plots. For % Uncertain Sleep Epochs, an operating point on the ROC curve was chosen where the specificity was ⩾ 95%, since a larger specificity for OSA patient detection was preferred. As such, a threshold of 64% was selected, at which specificity reached 97% and sensitivity 37%. For % Sleep Wake Transitions a threshold of 24% was selected, at which a specificity of 95% and sensitivity of 33% was obtained. AUROC, Area Under the ROC curve.

**Figure 5:**
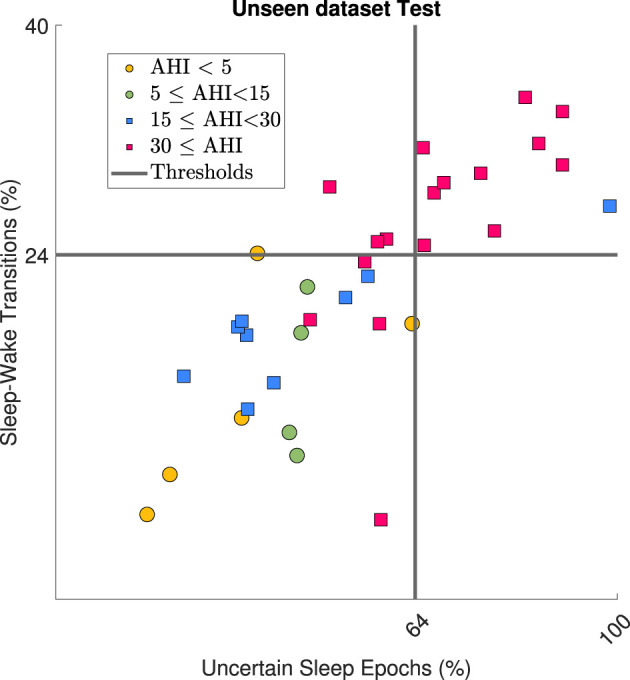
Application of two detection metrics on dataset Test where circles represent patients with AHI < 15 and squares AHI⩾15. Gray lines represent the corresponding metric thresholds. Severe OSA patients (AHI ⩾ 30) were detected correctly by the metrics, as expected.

The values of *No, Mild, Mod*, and *Sev* for % Uncertain Sleep Epochs and % Uncertain Wake Epochs increased with OSA severity class, as shown in Table 3. However, the trend was more pronounced for % Uncertain Sleep Epochs and was therefore chosen as the preferred metric. The distributions for *No, Mild, Mod*, and *Sev* with corresponding ROC curve for detection of AHI ⩾ 15 are displayed in Figure 4. The significance tests confirmed the increasing trend of % Uncertain Sleep Epochs with OSA severity. The area under the ROC curve was 0.77. Furthermore, an operating point on the ROC curve was chosen where the specificity was ⩾ 95%, since a larger specificity for detection of OSA patients was preferred. As such, a threshold of 64% was selected, at which specificity reached 97% and sensitivity 37%. A similar study was carried out for % Sleep Wake Transitions, for which the area under the ROC curve was 0.75. Also the upward trend with OSA severity was confirmed by a Kruskal–Wallis test (Figure 4). A threshold of 24% was selected, at which a specificity of 95% and sensitivity of 33% was obtained. The detection capabilities of these metrics and corresponding thresholds on Test are shown in Figure 5. Detection of OSA patients in set Test resulted in a κ of 32%, accuracy of 64%, sensitivity of 56%, and specificity of 89%. The specificity was relatively high, as expected, as there was only one false positive out of 36 patients.

## 4. Discussion

### 4.1. Sleep-Wake Classification

For sleep diagnostics of OSA patients in a home setting, sleep staging algorithms based on cardiac and respiratory signals are required, as these signals can be acquired by unobtrusive sensor technologies. However, many state-of-the-art sleep staging algorithms require long temporal dependencies in the data, which cannot be guaranteed in data acquired by unobtrusive sensors. Therefore, this study explicitly focussed on single short-term signal inputs for sleep staging. More specifically, this study proposed a deep learning network for sleep-wake classification based on single 30 s epochs from cardiac and respiratory signals in suspected OSA patients. Furthermore, the network was validated on an unseen test set.

The Wilcoxon signed rank tests showed that the RIP based network was more informative compared to the ECG based equivalent, as higher κ values were reached (see section 3.1). Nevertheless, application of the cardiac tachogram did have a benefit as combining the ECG and RIP signals into the multimodal network outperformed the RIP unimodal network. An additional advantage of including the cardiac tachogram is the usage of beat-to-beat variability, allowing the use of other cardiac sensors. Examples are pulse photoplethysmography and ballistocardiography, which enable heart beat extraction.

Furthermore, a distinction was made between epochs that reached prediction confidence thresholds and those which were uncertain. As reported in section 3.2, the % Uncertain Sleep Epochs *without* the presence of an apneic event was on average 30% of sleep predicted epochs. For these type of epochs, the prediction of N1 and REM epochs showed the lowest confidence. Both N1 and REM are more *active* stages of sleep, where the heart rate is elevated and the respiration more irregular (25, 26). This ressembles the cardiorespiratory behavior during wake and partially explains the larger confusion in prediction of these epochs. Furthermore, the ratio of N1 and REM epochs in the training data was low, as seen in Table 1. Hence, the network had less diverse examples to learn from, adding to the lower testing performances for N1 and REM epochs.

Comparison of the sleep-wake classification to literature was difficult as studies generally do not focus on using single short term epochs. Most studies include contextual information, by applying epoch sequences, which improves performance, at the cost of requiring long segments of good quality. This is extremely difficult to guarantee when using real and unobtrusive technology. Only the study of (12) fed single 30 s epochs from ECG to a CNN, but achieved a low κ of 0.25 for sleep-wake classification on healthy subjects. In contrast, the current study achieved a superior κ of 0.49 and 0.48 for mild and moderate OSA patients, respectively, which is in addition more challenging than classification in healthy subjects. On the other hand, (11) obtained a κ of 0.65 for wake-NREM-REM classification with pulse photoplethysmography in OSA patients with a median AHI of 16.8. Their performance was superior, but the used CNN was fed with a sequence of 100 epochs of 30 s. Similarly, (9) reached a κ of 0.62 for wake-NREM-REM using actigraphy and RIP in patients with an average AHI of 19.0. Their algorithm required a manual feature extraction on 25 epochs of 30 s. Although the current network reached lower κ scores compared to the latter studies, it offers a realistic approach for sleep-wake classification with unobtrusive sensors, as it is based on single 30 s epochs.

### 4.2. Total Sleep Time Estimation

The comparison of TST estimates with the reference value in Table 2 shows an increase in SD with an increase in AHI. It demonstrates a decrease in reliability of the outcome. Next, the estimation of TST on dataset Test was performed twice, once including all subjects (pre-detection) and once on subjects detection as non-OSA (post-detection). The reason for this was twofold. First, estimation of the TST becomes irrelevant when severe OSA patients can be detected, as they are directly prioritized for a clinical diagnostic test. Thus, an AHI estimation at home becomes redundant, as well as the corresponding TST estimation. Second, TST estimates becomes more reliable for milder OSA patients, due to more stable physiological dynamics, as further discussed in 4.3. For dataset Test, the ρ increased for post-detection (ρ = 0.74) compared to pre-detection (ρ = 0.46). Although the mean difference between the estimated TST and reference TST increased from − 9.7 min to − 21.9 min, the SD decreased from 101.0 to 55.7 min. Korkalainen et al. (11) reported a mean difference of − 12.2 min (±52.9 min) and (9) an overestimation of 14 min and ρ = 0.81. These studies performed slightly better on a population with a similar AHI range as expected, since their sleep staging performances were higher as well. Nevertheless, these studies required longer input intervals for the algorithm, making them less suited for usage on unobtrusive technologies. Moreover, this study slightly underestimated the TST, which would result in an overestimated AHI. In general, slight overestimation has minor consequences compared to underestimation, as these patients would receive a diagnostic PSG as a follow-up procedure.

### 4.3. Detection of OSA Patients

Despite the fact that the CNN was trained for sleep-wake classification, its outcome contained information relevant for detection of OSA patients. As discussed in section 2.4, more uncertain sleep-wake predictions were expected in the presence of apneic events, similar to the fact that the uncertainty of clinical sleep staging labels increases as well with the AHI of a patient. Additionally, there was an expected increase of sleep fragmentation, sympathetic activation and micro-awakenings related to apneas. As such, two metrics for detection of OSA patients were derived from the CNN outcome, namely the % Uncertain Sleep Epochs and % Sleep Wake Transitions. This improved interpretability of the network is beneficial when proposing the framework as a sleep diagnostics tool for OSA patients to clinicians. Another advantage was that OSA patient detection only relied on ECG and RIP signals, instead of including oxygen saturation sensors. A specificity of 89% was reached on the dataset Test, for detection of patients with AHI ⩾ 15. However, the corresponding sensitivity was only 56% and κ= 0.32. In addition, mainly severe OSA patients were detected, as illustrated in Figure 5. Indeed, when identifying an AHI ⩾ 30, the specificity remained stable at 89%, but the sensitivity increased to 78% and κ to 0.67. This result is beneficial, as severe OSA patient indeed require prioritization for diagnostic PSG at the hospital. Additionally, detection of patients with many events as a first step is advantageous for future refined OSA severity categorization. The reason is that severe OSA patients can have much stronger physiological dynamics compared to milder patients. This enables an OSA patient detection algorithm to focus training on patients with lower AHIs. It should be noted that one patient from Test with an AHI < 5 was falsely detected as being an OSA patient. For this, a follow-up over multiple nights could increase the OSA detection capabilities, as a single night recording might not be fully representative, due to accidental decreased data quality or the first night effect (27). If patients would consistently have values around the decision boundaries, it could indicate a pathological risk factor.

### 4.4. Future Work

To complete the proposed framework for OSA patient detection, apneic event detection from a minimal set of sensors is desired. This could be achieved by analyzing the SpO2 signal (28, 29) or the cardiac and respiratory signals, which are already included in the current sensor set (30, 31). Wearable trackers from several commercial companies already provide these signals, such as (32), (33), and (34). The number of apneic events could then be combined with the sleep-wake staging to calculate the patient's AHI and provide feedback on the OSA severity.

In addition, the algorithmic pipeline for sleep-wake classification and OSA patient detection requires further validation on unobtrusive data, as the presented study used PSG signals. This was partially performed by (13). However, this unobtrusive dataset was limited in number of subjects. Additionally, it only applied unobtrusively acquired ECG in combination with RIP from PSG. Thus, when accomodating the CNN to recordings from a different respiratory sensor, transfer learning of the new CNN is proposed. For this, the unimodal RIP network with the pretrained weights (see section 2.3.1) is updated with the new data using a very low learning rate. A small learning rate allows the model to learn an optimal set of weights. This retrained RIP network is then recombined into the multimodal network, after which the dense layers are retrained. A smaller number of subjects is required as the model was pretrained.

The presented framework could also benefit from training with a larger dataset to improve sleep-wake classification performance. Moreover, extending the problem to classes wake-NREM-REM could increase the relevance of the network and deliver insight into REM-related apneic events. These events are still being researched for their adverse effects on cardiac comorbidities (35, 36).

Furthermore, the application domain of confident epochs could be further extended. For example, the percentage of confident predicted epochs in a patient's recording could serve as a data quality indicator. In a sleep study recording patients over multiple nights, it is expected that this percentage would remain relatively stable for a subject. An outlier value could indicate a recording from different quality and instability of the percentage or a constant low percentage could even indicate sleep problems.

## 5. Conclusion

Standard clinical procedures for sleep monitoring rely on uncomfortable and burdensome EEG analysis. On the other hand, cardiac and respiratory signals have a great potential for comfortable sleep monitoring at home as unobtrusive sensors can record these. However, most unobtrusive sensors suffer from data loss and sensitivity to movement artifacts, especially in OSA patients. In addition, state-of-the-art sleep staging algorithms require long temporal dependencies, which cannot be garantueed in unobtrusive data. Therefore, this study developed a sleep-wake classifier to estimate the TST of suspected OSA patients based on single short-term (30 s) segments of their cardiac and respiratory signals. Application of the network on healthy, mild and moderate sleep apnea patients resulted in moderate κ scores of 0.51, 0.49, and 0.48. Furthermore, two metrics derived from the sleep-wake classifier's outcome were applied for detecting OSA patients in an unseen test set with patients of varying AHI. As such, severe OSA patients (AHI ⩾ 30) were detected in the unseen dataset with a sensitivity of 78% and specificity of 89%. Additional TST estimation was irrelevant for these detected patients, as they are directly prioritized for a clinical diagnostic test. Thus, their AHI estimation at home becomes redundant. Moreover, after excluding these severe patients, the overall accuracy of TST estimates increased to a mean bias error of 21.9 (± 55.7) min and Pearson correlation of 0.74 to the reference. As this patient detection was only based on cardiac and respiratory inputs, it might enable comfortable and fast prioritization of OSA patients for a diagnostic PSG. Overall, the presented framework offered a realistic tool for unobtrusive monitoring of sleep apnea patients.

## Data Availability Statement

The datasets presented in this article are not readily available because it contains information that could compromise the privacy of research participants and is subject to the European data-privacy policy regulations. The authors will try to provide an anonymized version of the dataset in compliance with the privacy policy of the University Hospitals of Leuven, which is the owner of the data. Requests to access the datasets should be directed to Carolina Varon, carolina.varon@esat.kuleuven.be.

## Ethics Statement

The studies involving human participants were reviewed and approved by ethical committee of UZ Leuven (S60319). The patients/participants provided their written informed consent to participate in this study.

## Author Contributions

DH and CV: conceptualization and methodology. DH: software, validation, formal analysis, investigation, visualization, and writing—original draft preparation. PB, DT, BB: resources and data curation. DH, PB, DT, BB, SV, and CV: writing—review and editing. SV and CV: supervision, project administration, and funding acquisition. All authors contributed to the article and approved the submitted version.

## Conflict of Interest

The authors declare that the research was conducted in the absence of any commercial or financial relationships that could be construed as a potential conflict of interest.

## Footnotes

^1^The network is publicly available at: https://github.com/dorienhsmns/TSTnet.

## Abbreviations

AHI: Apnea-Hypopnea Index
CNN: Convolutional Neural Network
OSA: Obstructive Sleep Apnea
ECG: Electrocardiography
EEG: Electroencephalogram
IHR: Instantaneous Heart Rate
N1, N2, N3: Non-Rapid Eye Movement sleep 1, 2, 3
PSG: Polysomnography
REM: Rapid Eye Movement
RIP: Respiratory Inductance Plethysmography
SD: Standard Deviation
TST: Total Sleep Time.

## References

[1] Young T Peppard PE Gottlieb DJ. Epidemiology of obstructive sleep apnea: a population health perspective. Am J Respir Crit Care Med. (2002) 165:1217–39. 10.1164/rccm.2109080 11991871

[2] Senaratna CV Perret JL Lodge CL Lowe AJ Campbell BE Matheson MC. Prevalence of obstructive sleep apnea in the general population: a systematic review. Sleep Med Rev. (2017) 34:70–81. 10.1016/j.smrv.2016.07.002 27568340

[3] Flemons WW Douglas NJ Kuna ST Rodenstein DO Wheatley J. Access to diagnosis and treatment of patients with suspected sleep apnea. Am J Respir Crit Care Med. (2004) 169:668–72. 10.1164/rccm.200308-1124PP 15003950

[4] Berry RB Budhiraja R Gottlieb DJ Gozal D Iber C Kapur VK. Rules for scoring respiratory events in sleep: update of the 2007. AASM manual for the scoring of sleep and associated events. J Clin Sleep Med. (2012) 8:597–619. 10.5664/jcsm.2172 23066376 PMC3459210

[5] Sateia MJ. International classification of sleep disorders. Chest. (2014) 146:1387–94. 10.1378/chest.14-0970 25367475

[6] Rechtschaffen A Kales A. A Manual of Standardized Terminology, Techniques, and Scoring System for Sleep Stages for Human Subjects. National Institute of Health (1968).11422885

[7] Willemen T Varon C Caicedo Dorado A Haex B Vander Sloten J Van Huffel S. Probabilistic cardiac and respiratory based classification of sleep and apneic events in subjects with sleep apnea. Physiol Meas. (2015) 36:2103. 10.1088/0967-3334/36/10/2103 26290159

[8] Radha M Fonseca P Moreau A Ross M Cerny A Anderer P. Sleep stage classification from heart-rate variability using long short-term memory neural networks. Sci Rep. (2019) 9:14149. 10.1038/s41598-019-49703-y 31578345 PMC6775145

[9] Dietz-Terjung S Martin AR Finnsson E Ágústsson JS Helgason S Helgadóttir H. Proof of principle study: diagnostic accuracy of a novel algorithm for the estimation of sleep stages and disease severity in patients with sleep-disordered breathing based on actigraphy and respiratory inductance plethysmography. Sleep Breath. (2021) 1–8. 10.1007/s11325-021-02316-0 33594617 PMC8590674

[10] Bakker JP Ross M Vasko R Cerny A Fonseca P Jasko J. Estimating sleep stages using cardiorespiratory signals: validation of a novel algorithm across a wide range of sleep-disordered breathing severity. J Clin Sleep Med. (2021). 10.5664/jcsm.9192. [Epub ahead of print]. 33660612 PMC8314617

[11] Korkalainen H Aakko J Duce B Kainulainen S Leino A Nikkonen S. Deep learning enables sleep staging from photoplethysmogram for patients with suspected sleep apnea. Sleep. (2020) 43:zsaa098. 10.1093/sleep/zsaa098 32436942 PMC7658638

[12] Malik J Lo YL Wu H. Sleep-wake classification via quantifying heart rate variability by convolutional neural network. Physiol Meas. (2018) 39:085004. 10.1088/1361-6579/aad5a9 30043757

[13] Huysmans D Heffinck E Castro I Deviaene M Borzee P Buyse B. Sleep-wake classification for home monitoring of sleep apnea patients. In: Proceedings of the 47th Annual Computing in Cardiology Conference. Rimini: IEEE (2020). 10.22489/CinC.2020.147

[14] Norman RG Pal I Stewart C Walsleben JA Rapoport DM. Interobserver agreement among sleep scorers from different centers in a large dataset. Sleep. (2000) 23:901–8. 10.1093/sleep/23.7.1e 11083599

[15] Varon C Van Huffel S. Complexity and nonlinearities in cardiorespiratory signals in sleep and sleep apnea. In: Barbieri R Scilingo EP Valenza G, editors. Complexity and Nonlinearity in Cardiovascular Signals. Cham: Springer (2017). p. 503–37. 10.1007/978-3-319-58709-7_19

[16] Fonseca P van Gilst MM Radha M Ross M Moreau A Cerny A. Automatic sleep staging using heart rate variability, body movements, and recurrent neural networks in a sleep disordered population. Sleep. (2020) 43:zsaa048. 10.1093/sleep/zsaa048 32249911

[17] Medatec. Medatec. (2021). Available online at: https://www.medatec.eu/en/sleep

[18] Moeyersons J Amoni M Van Huffel S Willems R Varon C. R-DECO: An open-source Matlab based graphical user interface for the detection and correction of R-peaks. PeerJ Comput Sci. (2019) 5:e226. 10.7717/peerj-cs.226 PMC7924703 33816879

[19] Pichot V Roche F Celle S Barthélémy JC Chouchou F. HRVanalysis: a free software for analyzing cardiac autonomic activity. Front Physiol. (2016) 7:557. 10.3389/fphys.2016.00557 27920726 PMC5118625

[20] Srivastava N Hinton G Krizhevsky A Sutskever I Salakhutdinov R. Dropout: a simple way to prevent neural networks from overfitting. J Mach Learn Res. (2014) 15:1929–58.

[21] Kingma DP Ba J. Adam: a method for stochastic optimization. arXiv preprint arXiv:14126980. (2014).

[22] McHugh ML. Interrater reliability: the kappa statistic. Biochem Med. (2012) 22:276–82. 10.11613/BM.2012.031 PMC3900052 23092060

[23] Kimoff RJ. Sleep fragmentation in obstructive sleep apnea. Sleep. (1996) 19:S61–6. 10.1093/sleep/19.suppl_9.S61 9122574

[24] Guilleminault C Winkle R Connolly S Melvin K Tilkian A. Cyclical variation of the heart rate in sleep apnoea syndrome: mechanisms, and usefulness of 24 h electrocardiography as a screening technique. Lancet. (1984) 323:126–31. 10.1016/S0140-6736(84)90062-X 6140442

[25] Douglas NJ White DP Pickett CK Weil JV Zwillich C. Respiration during sleep in normal man. Thorax. (1982) 37:840–4. 10.1136/thx.37.11.840 7164002 PMC459437

[26] Bassetti C Dogas Z Peigneux P. Sleep Medicine Textbook. Regensburg: European Sleep Research Society (2014).

[27] Agnew H Jr. Webb WB Williams RL. The first night effect: an EEG studyof sleep. Psychophysiology. (1966) 2:263–6. 10.1111/j.1469-8986.1966.tb02650.x 5903579

[28] Deviaene M Testelmans D Buyse B Borzée P Van Huffel S Varon C. Automatic screening of sleep apnea patients based on the spo 2 signal. IEEE J Biomed Health Inform. (2018) 23:607–17. 10.1109/JBHI.2018.2817368 29993790

[29] Mendonça F Mostafa SS Morgado-Dias F Ravelo-Garcia AG. An oximetry based wireless device for sleep apnea detection. Sensors. (2020) 20:888. 10.3390/s20030888 32046102 PMC7039040

[30] Feng K Qin H Wu S Pan W Liu G. A Sleep apnea detection method based on unsupervised feature learning and single-lead electrocardiogram. IEEE Trans Instrument Meas. (2020) 70:1–12. 10.1109/TIM.2020.3017246

[31] Deviaene M Castro I Borzée P Patel A Torfs T Buyse B. Capacitively-coupled ECG and respiration for the unobtrusive detection of sleep apnea. Physiol Meas. (2021) 42:024001. 10.1088/1361-6579/abdf3d 33482650

[32] Fitbit. Fitbit SpO2. (2021). Available online at: https://www.fitbit.com/global/us/technology/health-metrics

[33] Garmin. Garmin SpO2. (2021). Available online at: https://www.garmin.com/en-US/

[34] Apple. Apple SpO2. (2021). Available online at: https://www.apple.com/watch/

[35] Aurora RN Crainiceanu C Gottlieb DJ Kim JS Punjabi NM. Obstructive sleep apnea during REM sleep and cardiovascular disease. Am J Respir Crit Care Med. (2018) 197:653–60. 10.1164/rccm.201706-1112OC 29112823 PMC6005240

[36] Varga AW Mokhlesi B. REM obstructive sleep apnea: risk for adverse health outcomes and novel treatments. Sleep Breath. (2019) 23:413–23. 10.1007/s11325-018-1727-2 30232681 PMC6424642

