# Supplementary material for: Sleep Diagnostics for Home Monitoring of Sleep Apnea Patients
Source: Front Digit Health. 2021 Jun 15;3:685766. doi: 10.3389/fdgth.2021.685766 (PMC8521961; doi:10.3389/fdgth.2021.685766)
Supplement: Supplementary file 1 [file Table_1.pdf]

		Prediction (%)	
		**Sleep**	**Wake**
**Ground Truth**	**N3**	3.1	1.7
	**N2**	10.6	6.8
	**N1**	2.3	2.8
	**REM**	10.0	7.8
	**Wake**	5.7	19.1

Table 1. To investigate the cause of uncertainty for non-apneic epochs, the ground truth sleep stages of these epochs were extracted for $\mathrm{CNN\_Test}$. The percentage indicates the **ratio of uncertain non-apneic epochs to the total number of epochs**. The largest portion of uncertain sleep predicted, non-apneic epochs were found during N2 and REM sleep as indicated in grey.
