# Supplementary material for: Sleep Diagnostics for Home Monitoring of Sleep Apnea Patients
Source: Front Digit Health. 2021 Jun 15;3:685766. doi: 10.3389/fdgth.2021.685766 (PMC8521961; doi:10.3389/fdgth.2021.685766)
Supplement: Supplementary file 2 [file Table_2.pdf]

		Prediction (%)		
		**Sleep**	**Wake**	$\sum$
**Ground Truth**	**N3**	12.0	0.9	12.9
	**N2**	21.9	1.9	23.8
	**N1**	46.0	9.1	55.1
	**REM**	48.9	4.3	53.2
	**Wake**	33.9	16.8	50.7

$\sum$: Summation

Table 2. To investigate the cause of uncertainty for non-apneic epochs, the ground truth sleep stages of these epochs were extracted for $\mathrm{CNN\_Test}$. The percentage indicates the **ratio of uncertain non-apneic epochs to the number of epochs in a specific sleep stage**. Thus, the right column $\sum$ is the total ratio of uncertain non-apneic epochs per sleep stage. It is observed that the classes N1 and REM had the largest ratio of uncertain non-apneic epochs, being 55.1% and 53.2%, respectively (indicated in grey). However, uncertain predictions did not necessarily imply incorrect predictions. Nevertheless, classes N1 and REM also had the largest ratio of uncertain non-apneic epochs which were wrongly predicted, respectively 9.1% and 4.3% (indicated in grey).
